# The Current Status of Microscopical Hair Comparisons

**DOI:** 10.1100/tsw.2001.358

**Published:** 2001-12-08

**Authors:** Walter F. Rowe

**Affiliations:** Department of Forensic Sciences, The George Washington University, Washington, DC 20052, USA

**Keywords:** comparative microscopy, hair, hair comparisons, microscopy, mitochondrial DNA, mtDNA

## Abstract

Although the microscopical comparison of human hairs has been accepted in courts of law for over a century, recent advances in DNA technology have called this type of forensic examination into question. In a number of cases, post-conviction DNA testing has exonerated defendants who were convicted in part on the results of microscopical hair comparisons. A federal judge has held a Daubert hearing on the microscopical comparison of human hairs and has concluded that this type of examination does not meet the criteria for admission of scientific evidence in federal courts. A review of the available scientific literature on microscopical hair comparisons (including studies conducted by the Royal Canadian Mounted Police and the Federal Bureau of Investigation) leads to three conclusions: (1) microscopical comparisons of human hairs can yield scientifically defensible conclusions that can contribute to criminal investigations and criminal prosecutions, (2) the reliability of microscopical hair comparisons is strongly affected by the training of the forensic hair examiner, (3) forensic hair examiners cannot offer estimates of the probability of a match of a questioned hair with a hair from a randomly selected person. In order for microscopical hair examinations to survive challenges under the U.S. Supreme Court’s Daubert decision, hair microscopists must be better trained and undergo frequent proficiency testing. More research on the error rates of microscopical hair comparisons should be undertaken, and guidelines for the permissible interpretations of such comparisons should be established. Until these issues have been addressed and satisfactorily resolved, microscopical hair comparisons should be regarded by law enforcement agencies and courts of law as merely presumptive in nature, and all microscopical hair comparisons should be confirmed by nuclear DNA profiling or mitochondrial DNA sequencing.

## INTRODUCTION

The technology of microscopic hair comparisons has not changed significantly for many decades. In such comparisons, a questioned hair is compared with known hair samples using a transmitted-light comparison microscope. The following features of the questioned hair and each of the known hairs are compared[1]:

1. Color (which includes the basic hue of the hairs, pigment density, pigment distribution, pigment aggregation, pigment size, pigment shape, and pigment color)
2. Structure (which includes the form, diameter and diameter variation, cross-sectional shape, cortical structure, medullation, and the presence or absence of shaft aberrations)
3. Cuticular traits (which include the size and shape of the cuticular scales and the appearance of the inner and outer margins of the cuticle)
4. Acquired characteristics (such as cosmetic treatments, cleanliness, abnormalities, and artifacts)

Ideally, the forensic hair examiner will find a one-to-one correspondence between the features of the questioned hair and those of at least one of the known hairs, with no significant differences along the lengths of the hairs. Generally, a forensic hair examiner will render one of three conclusions: (1) the questioned and known hairs "match" (i.e., display this one-to-one correspondence), and, therefore, a possible association exists between the questioned hair and the source of the known hairs; (2) the hairs do not match and do not have a common origin; or (3) no conclusion can be drawn from the microscopic hair comparisons[1]. The basic principles of the microscopic examination of human hairs have been presented by Hicks[2], Seta et al.[3], Deedrick[4], and Bisbing[1].

The microscopical comparison of hairs has been used in criminal investigations and criminal prosecutions for over a century. During this period courts of law routinely admitted the results of microscopical comparisons. However, the development of DNA technology has led to a reevaluation of this type of forensic analysis. DNA profiling has permitted convicted defendants to have biological evidence reexamined; such reexaminations have resulted in a number of convictions being overturned. In 1996 the National Institute of Justice published the results of a study of postconviction reversals of convictions based on new DNA tests[5]. This study identified 27 criminal cases in which postconviction DNA testing had produced exculpatory evidence that led either to new trials or to pardons for defendants. In six of these cases, the results of microscopical hair comparisons had formed a significant part of the prosecution's evidence. Five of these six cases are the most infamous miscarriages of justice corrected by DNA profiling: those of Gary Dotson, Ricky Hammond, Edward Honaker, David Vasquez, and Glen Woodall. The authors of this study made the following comments vis-à-vis non-DNA evidence:

> In many of the study cases, according to documentation examined and those interviewed, scientific experts had convinced juries that non-DNA analyses of blood or hair were reliable enough to clearly implicate the defendants. Scientific evidence based on non-DNA analyses, however, has proven less discriminating and reliable than those based on DNA tests. [5, p. 25]

Later studies have added to the list of cases involving hair comparison evidence in which verdicts have been overturned by postconviction DNA testing. *Actual Innocence*, a recent book by Scheck, Neufeld, and Dwyer, characterized microscopical hair comparisons as junk science, and added the cases of Ron Williamson and Dennis Fritz and of Timothy Durham to the list of cases in which microscopical hair comparisons were shown to have yielded erroneous results[6]. As befits high-profile defense attorneys, the authors of *Actual Innocence* were scathing in their assessment of microscopical hair comparisons:

> After a century of expert testimony about microscopic hair comparisons, DNA tests had exposed the field as lethal nonsense: 29 percent of the wrongful convictions studied by the Innocence Project included evidence from hair analyses.[6, p. 166]

A more dispassionate evaluation of these cases reveals a more complicated picture. The evidence against the defendants in these cases did not consist solely of hair comparisons[5]. In the *Dotson* case, for example, the prosecution's case against Gary Dotson on charges of aggravated kidnapping and rape included testimony that a pubic hair removed from the complainant's underwear was microscopically similar to samples of Dotson's pubic hair but microscopically dissimilar to the complainant's own pubic hair. The complainant also made a perjurious identification (recanted 6 years later[7]) of Dotson as her attacker, and the prosecution's expert serologist testified that the semen on the complainant's underwear came from a type-B secretor (which Dotson was determined to be). The serologist somehow failed to inform the court that the complainant was also a type-B secretor[5]. The serological results were consistent with the semen coming from a type-B secretor, a type-O secretor, or a nonsecretor of any ABO blood type. In the *Vasquez* case, the prosecution proffered both the results of a microscopical hair comparison which found that pubic hairs recovered at the crime scene were consistent with the defendant's pubic hairs and the testimony of two witnesses who placed the defendant near the rape-murder victim's house on the day of the crime. The mentally retarded defendant entered an Alford plea to second-degree homicide and burglary to avoid standing trial on a capital murder charge.

Nevertheless, the impact of microscopical hair comparisons on criminal convictions cannot be exaggerated. A comprehensive study by the *Chicago Tribune*[8] of death-penalty cases in Illinois, since the reinstatement of the death penalty in 1977, revealed that prosecutors *relied* on microscopical hair comparisons in at least 20 of the 285 cases in which the defendant was sentenced to death. The *Chicago Tribune* study found that, overall, hair examinations played a major role in half of the death penalty cases.

## A *DAUBERT* CHALLENGE

Prior to 1993, the precedent decision governing the admission of scientific evidence in most federal courts was *Frye v. United States*[9]. This appellate decision arose from the attempt of the defendant in a murder case in the District of Columbia to introduce the results of an allegedly exculpatory polygraph ("lie detector") test. After his conviction, the defendant appealed the trial court's refusal to admit the polygraph test results. The D.C. Court of Appeals upheld the exclusion of the polygraph results, stating the following:

> Just when a scientific principle or discovery crosses the line between the experimental and demonstrable stages is difficult to define. Somewhere in this twilight zone the evidential force of the principle must be recognized, and while courts will go a long way in admitting expert testimony deduced from a well recognized scientific principle or discovery, the thing from which the deduction is made must be sufficiently established to have gained general acceptance in the particular field in which it belongs.[9]

In 1993 the U.S. Supreme Court decided the case of *Daubert v. Merrell Dow Pharmaceuticals*[10]. In this case, the Supreme Court held that Rule 702 of the Federal Rules of Evidence overruled the *Frye* decision and the majority opinion provided a set of guidelines for federal judges to apply in determining whether a theory or technique is scientific:

1. Can the theory or technique be tested? Has it been so tested?
2. Has the theory or technique been subjected to peer review and been published?
3. What is the known or potential error rate?
4. Are there standards controlling the technique's operation?
5. Does the theory or technique have widespread acceptance within the relevant scientific community?

Although this decision establishes general rules for admission of scientific evidence only for federal courts, courts in other jurisdictions pay careful attention to decisions of the U.S. Supreme Court.

In 1995 a federal judge applied the criteria set out in *Daubert* to hair examinations in *Williamson v. Reynolds*[11]. Ron Williamson and Dennis Fritz were convicted and condemned to death in Oklahoma for a rape and murder. At their trials evidence from a hair examiner was presented that 13 hairs found on the victim could have come from Fritz and 4 others could have come from Williamson. Fritz was convicted and sentenced to life in prison, while Williamson was sentenced to death. Williamson sought a writ of habeas corpus in federal court, challenging his conviction on a number of grounds. Among them was the contention that the trial court erred in admitting the testimony of the hair examiner. The federal district court granted Williamson's writ of habeas corpus and concluded that the state's hair evidence did not meet the *Daubert* standard. The court's comments on the qualifications of those forensic scientists conducting microscopical hair comparisons were unsparing:

> Not even the "general acceptance" standard is met, since any "general acceptance"seems to be among hair experts who are generally technicians testifying for the prosecution, not scientists who can objectively evaluate such evidence.

This part of the federal district court decision was overturned on appeal on the ground that federal courts should apply a due-process/fundamental-fairness standard in evaluating scientific evidence admitted by state courts[12].[a] *Williamson v. Reynolds* has become the centerpiece of attacks on the admissibility of the results of forensic identification testimony generally[13].

## FLAWS IN FORENSIC HAIR COMPARISON EVIDENCE

Only in the case of Guy Paul Morin has there been a public accounting of what went wrong with the hair comparison evidence[14]. In 1992 Morin was convicted in Ontario, Canada, after two trials of the 1984 murder of Christine Jessop. Part of the evidence presented against Morin was a hair fragment found on the victim's necklace: the prosecution's expert testified that she could not exclude Morin as the source of this hair. This expert also testified that three hairs found in Morin's Honda car were dissimilar to Morin's hairs, and could have come from the victim. All of the questioned hairs had undergone significant deterioration prior to being collected as evidence; consequently, the prosecution's expert could not state that the questioned hair specimens were consistent with known samples from Jessop or Morin. Morin was convicted and had served several years in prison when a DNA test of the semen left by the murderer on the victim's panties revealed that Morin was not the source of the semen. Morin was exonerated and the government of Ontario created a commission to "inquire into the conduct of the investigation into the death of Christine Jessop, the conduct of the Centre of Forensic Sciences in relation to the maintenance, security, and preservation of forensic evidence, and into the criminal proceedings involving the charge that Guy Paul Morin murdered Christine Jessop." After a lengthy series of hearings, the commission issued a scathing indictment of the conduct of virtually everyone involved on the prosecution's side of the case: the police investigators, the forensic experts, and even the parents of the murder victim (whose perjury at trial was suborned by police). A critical issue in the inquiry was the communication of the results of the microscopical hair comparisons to police

[a] This case could serve as a prime example of the inadequacies of the microscopic comparison of hair that have been exposed by postconviction DNA testing. In 1999 mitochondrial DNA testing was conducted on the 17 hairs from the crime scene that had been microscopically 'matched' to Williamson and Fritz: mitochondrial DNA sequencing eliminated both Williamson and Fritz as the sources of these hairs. At the time of the original investigation, microscopic hair comparisons had eliminated a man named Glen Gore as a source of any of the crime scene hairs. At their trials, Gore was an important prosecution witness against both Williamson and Fritz. The mitochondrial DNA sequencing of the hairs linked Gore to two of the hairs from the murder victim's body[6].

investigators during the initial investigation: the hair expert claimed to have conveyed to police investigators the limitations of her test results. The commission report commented on this matter:

> I find that [the prosecution's hair expert] did conduct a hasty, preliminary comparison of the necklace hair and Guy Paul Morin's hairs in the officers' presence. She did communicate a preliminary opinion to the officers. That opinion, however, was overstated and, to her knowledge, left the officers with the understanding that the comparison yielded important evidence implicating Mr. Morin[14].

The report concluded with the following recommendation with regard to hair comparison evidence:

> Trial judges should undertake a more critical analysis of the admissibility of hair comparison evidence as circumstantial evidence of guilt. Evidence that shows only that an accused cannot be excluded as the donor of an unknown hair (or only that an accused may or may not have been the donor) is unlikely to have sufficient probative value to justify its reception at a criminal trial as circumstantial evidence of guilt[14].

Recently, a major investigation has been launched into the conduct of Joyce Gilchrist, a forensic chemist in the Oklahoma City police crime laboratory[15]. Ms. Gilchrist had been cited by the Oklahoma Court of Criminal Appeals for overstating the results of her microscopical hair comparisons. In a typical scenario, she would state that hair comparisons do not provide an absolute means of personal identification and then she would go on to state that based on her examinations the defendant(s) had been in contact with the victim or the crime scene[16,17]. The FBI reviewed eight of her cases and concluded that Ms. Gilchrist had overstated the value of her microscopical hair examinations in five of them[18]. All of her casework is currently under review, including a number of capital cases in which the defendants have been executed[19]. How adequate this investigation will be remains to be seen: one of the hair examiners participating in the investigation is the hair examiner who conducted the hair comparisons in the *Fritz* and *Williamson* cases[20].

## THE ROYAL CANADIAN MOUNTED POLICE (RCMP) STUDIES

The known or potential error rate of a scientific technique is one of the admissibility criteria set forth in the *Daubert* decision. A number of studies have been performed to determine the error rate of microscopical hair comparisons. The studies have been structured in a variety of ways: in some the examiners have known whether the hairs being compared microscopically shared a common source or not; and in others the examiners have been kept ignorant of the sources of the hairs. In 1974 Gaudette and Keeping[21] published the results of study of the microscopical comparisons of human head hair. This study was conducted as follows:

- 100 individuals provided 80 to 100 hairs randomly selected from various parts of the scalp.
- 6 to 11 dissimilar hairs were chosen from each sample. These subsamples were examined microscopically and their microscopic features coded.
- Hairs with similar codes were compared microscopically.
- Of 861 hairs from 100 individuals, 9 pairs were found to be indistinguishable.
- The authors calculated the odds of one head hair randomly selected from one individual being indistinguishable from a head hair randomly selected from a second individual to be 1/4500.

This study was followed by a second study of the microscopical comparisons of human pubic hair[22]. This study was structured in a manner similar to the previous one:

- 30 hairs were plucked from each of 60 individuals.
- 6 to 11 dissimilar hairs were selected from each sample; these were examined microscopically and their microscopic features coded.
- Hairs with similar codes were then compared microscopically.
- The author calculated the probability of a single pubic hair randomly selected from one individual being indistinguishable from a pubic hair randomly selected from a second individual to be 1/800.

These studies are open to criticism on a number of grounds. First of all, because virtually all of the contributors of the hairs in the two studies were Caucasians, the probability estimates, if valid, apply only to Caucasians and cannot be reliably extrapolated to hair from persons with other population ancestries. Second, the comparisons were not blind: the examiners were aware whether hairs being compared were from the same source or not. This could lead to unconscious biasing of results. Finally, the selection of the subsamples used in the microscopical comparisons was not random. Following the publication of these studies a controversy raged about what question microscopical hair comparisons are intended to answer and whether these two studies in fact answered the correct question[23,24]. Aitken and Robertson[25] have pointed out that, leaving such contentious issues aside, the studies of Gaudette and Keeping[21] and Gaudette[22] only provide *average* probability estimates (i.e., estimates that cannot be applied to any particular hair comparison) and beyond that the probability estimates apply only to the examiners at the RCMP forensic laboratory where the research was conducted.

## BLIND STUDIES

Wickenheiser and Hepworth[26] attempted to replicate the work of Gaudette and Keeping[21], while eliminating the likelihood of examiner bias. In their study, 100 or more scalp hairs were taken from 100 Causasians. From each of these hair samples, 5 to 13 macroscopically dissimilar hairs were selected for microscopical examination. An independent party randomly numbered the dissimilar hairs and also added an additional 53 randomly selected hairs to those to be examined microscopically. To reduce the number of potential microscopical comparisons the two examiners conducting the study each separately categorized the hairs using 14 characteristics: color, 7 major characteristics (treatment , texture, pigment distribution, medulla, medullary index, maximum diameter, and the presence of cortical fusi), and 6 secondary characteristics (length, cuticular margin, tip characteristics, pigment density, pigment size, and shaft diameter variation). Hairs selected for microscopical comparisons had to have the same-color classification and the same major characteristics; small variations in the secondary characteristics were permitted. Two hairs were determined to match if they shared all characteristics in common, including the variation of these characteristics from root to tip. The two hair examiners working independently found seven and six one-to-one matches. However, due to differences in the ways that the two examiners categorized the hairs, there was only one one-to-one match in common. In no case was a hair from one individual incorrectly matched to that of another. The authors of this study also concluded that the macroscopic selection of a small number of dissimilar hairs from a larger sample did not ensure an adequate representation of the range of microscopic features in the known hair samples.

The Forensic Science Service in Great Britain conducted a collaborative hair comparison study involving forensic science laboratories in the U.K. and in North America[27]. Although eight laboratories initially agreed to participate, the time required to circulate and examine the hair specimens proved to be so great that all but four laboratories (two in North America and two in the U.K.) dropped out of the study. The samples examined were pigmented Caucasian hairs obtained from postmortem examinations. A single hair from each subject was mounted on a microscope slide; the mounted hairs were designated the questioned hairs. Ten loose hairs were provided as known hairs. The participating forensic hair examiners all examined the same questioned and known specimens. The study was structured so that one questioned hair sample had no matching known hairs and two of the sets of known hairs had no matching questioned hairs. Two of the questioned hairs were from the same source. The forensic hair examiners were asked to address the question: "Has the questioned hair come from the same source as the known sample?" In contrast to what would occur in actual casework (in which the examiner would merely be asked to indicate whether a questioned and known hair matched), the participating examiners were asked to indicate the strength of their opinion using a numerical scale, viz.:

- +5: I am sure the answer is yes (i.e., the questioned and known hairs came from the same source).
- +4: I am almost sure the answer is yes.
- +3: Very probably yes.
- +2: Probably yes.
- +1: Possibly yes.
- 0: I have not the vaguest idea.
- -1: Possibly no.
- -2: Probably no.
- -3: Very probably no.
- -4: I am almost sure the answer is no.
- -5: I am sure the answer is no[27].

Overall scores for each participating laboratory were determined by giving each correct match or exclusion a positive value equal to the level of certainty and each incorrect match or exclusion a negative value equal to the level of certainty. For example, a participating laboratory, which correctly matched every questioned sample to its known hair sample with the highest level of confidence and correctly excluded every questioned and known hair samples having different origins with the same level of confidence, would have a score of 500. Theoretically, a novice hair examiner with no training or experience in hair comparisons would score a zero (have no basis for a determination). One laboratory achieved a score of 493 (98% of the maximum attainable score), while the other laboratories achieved, respectively, 60, 46, and 43% of the maximum attainable score. The author of the study tied the differing performance levels to different standards of training. The high-scoring forensic laboratory "holds intensive training courses, organised and taught by very experienced hair examiners." In the low-scoring laboratories (both in the U.K.), on the other hand, "current training is an ad hoc, hit or miss affair."

## DNA PROFILING

DNA technology has been extended to the examination of hairs[28,29,30,31,32,33,34]. Hair follicle tags (avulsed linings of hair follicles) have proven to be a good source of nuclear DNA, as have the roots of growing hairs (anagen-phase hairs or anagen hairs progressing to the catagen phase). Unfortunately, most of the hairs encountered in casework are telogen-phase hairs, whose roots are completely keratinized. Attempts have been made to extract, amplify, and type nuclear DNA from telogen hairs with only limited success. The extraction and sequencing of mitochondrial DNA (mtDNA) from telogen hairs have been much more successful. Mitochondria are the organelles in cells that produce energy; each cell contains hundreds or thousands of mitochondria. Each mitochondrion contains several copies of mtDNA. Thus, typically each cell in the body contains thousands of copies of mtDNA, but only a single copy of nuclear DNA. The large numbers of copies of mtDNA make it easier to reliably extract and sequence mtDNA than to extract and type nuclear DNA[1].

Mitochondrial DNA consists of a loop of DNA containing approximately 16,600 base pairs loop. Two regions of the loop, designated Hypervariable Region I (HV1) and Hypervariable Region II (HV2), show significant variation in the human population and are the targets of mtDNA sequencing. Variations in the sequences of these two regions have been compiled into databases. Unfortunately, the present mtDNA databases are too small to permit meaningful estimates of the frequency of a particular mtDNA sequence in the human population. At best, a laboratory performing mtDNA sequencing on hairs can report the number of times that the mtDNA sequence found in the hairs occurs in the database. Given the limited number of base pairs in mtDNA, it is unlikely that mtDNA sequencing will ever be as discriminating as nuclear DNA profiling. Therefore, microscopical hair comparisons, if valid, could make substantial contributions to forensic hair examinations[1].

Linch et al.[34] recently published a review of hair developmental biology for forensic scientists performing mtDNA analyses. The authors made the following comments regarding microscopical hair comparisons:

> … [C]omparison transmitted light microscopy remains the most timely and cost effective method to determine which hairs in a case should be referred for possible DNA typing[34].

The authors recommended that microscopical biological profiles of evidentiary hairs should be prepared, including such information as species identification, somatic origin, population ancestry, and root-morphology classification. They also cautioned hair microscopists to keep in mind the possibility of false exclusions before rejecting mtDNA analysis.

## COMPARISON OF DNA ANALYSES WITH MICROSCOPICAL HAIR COMPARISONS

The FBI has conducted a retrospective study of all human hair examinations submitted to its laboratory for mitochondrial DNA analysis between 1996 and 2000[35]. In the FBI laboratory, a microscopical hair comparison can yield one of three possible outcomes: a positive association (the questioned and known hairs could have a shared a common origin), a negative association (the questioned and known hairs did not have a common origin), and an inconclusive result. A positive association is reviewed by a second examiner, and if the second examiner concurs with this conclusion, the questioned hair is then subjected to mtDNA analysis. In less than 9% of the cases in which FBI hair examiners found a positive association did the mtDNA analysis yield an exclusion. The authors of this study concluded the following:

> The correlation between microscopic hair comparison results and mtDNA analysis results is good, indicating a solid foundation for the genetic and scientific basis of microscopic hair comparisons[35].

## HAIR TRANSFER

Regardless of whether hairs are compared microscopically or are submitted to mtDNA sequencing, the ultimate significance of the analyses depends on the context in which the questioned hair was found. This may be illustrated by the following scenarios:

- Scenario A: A woman is found raped and murdered in her apartment. A questioned pubic hair found in the victim’s pubic area is associated with a suspect. When questioned by police, the suspect denies ever having contact with the victim or ever being in her apartment.

- Scenario B: A woman is found raped and murdered in her apartment. A questioned head hair recovered from vacuum sweepings of the apartment is associated with a suspect. The suspect is a former boyfriend of the victim and has lived with her off and on for a number of years.

In scenario A, the hair evidence is particularly strong: the most parsimonious explanation for the association of the pubic hair is that it was transferred from the suspect to the victim when he assaulted her. The type of hair and its location make a casual transfer between the suspect and the victim (say, due to them brushing against one another on the street or on public transportation) unlikely. In scenario B, the hair evidence has no probative value. Most people shed up to a hundred head hairs each day; consequently, the suspect might have left behind hundreds of his head hairs in the victim's apartment during the time he lived there.

In most forensic cases the probative value of hair evidence falls between the extremes of the two scenarios above. In order to assess completely the significance of a hair association in a particular case, the forensic hair examiner should have some familiarity with the transfer of hairs and their persistence on clothing and other surfaces. Forensic science has only a few general principles; one of them is the Locard Exchange Principle. This principle states that when two surfaces come into contact, material is exchanged across the interface[36]. There are several types of transfers: in a *primary* transfer material is transferred from A to B and is recovered from B; in a *secondary* transfer material is transferred from A to B, then from B to C, and is recovered from C.

Gaudette and Tessarolo[37] have reported a series of experiments involving the secondary transfer of human scalp hair. They also examined the persistence of hairs: fluorescently dyed hairs were placed on the outer clothing of ten volunteers in a forensic science laboratory, and the locations of the hairs were determined with a UV lamp at various times during the work day. After 8 h, just 6% of the hairs remained on the volunteers' clothing. The decay curve for the persistence of hairs determined in this study was very similar to that found for the persistence of textile fibers.

In the *Morin* case, the police investigators and the forensic hair examiner may have given insufficient consideration to the context in which matching hairs were found[14]. Morin lived with his family next door to the Jessops. The Morins and Jessops had been in and out of each others' homes and vehicles, both before and after Christine Jessop's abduction and murder. It was certainly conceivable that mutual transfers of hairs could have occurred prior to Morin becoming a suspect in the case.

## CONCLUSION

A review of the available scientific studies of microscopical hair comparisons leads to the following conclusions:

1. Despite assertions to the contrary, microscopical comparisons of human hairs can yield scientifically defensible conclusions that can contribute to criminal investigations and criminal prosecutions, even in the era of DNA analysis.
2. The reliability of microscopical hair comparisons is strongly affected by the training of the forensic hair examiner.
3. Forensic hair examiners cannot at present offer estimates of the probability of a match of a questioned hair with a hair from a randomly selected person.

If microscopical hair comparisons are to survive future *Daubert* challenges, a number of issues would have to be addressed:

1. Training of forensic hair microscopists: the training of hair microscopists has been shown to be a critical determinant of the accuracy of the microscopical hair comparisons they perform. The Scientific Working Group on Materials Analysis (SWGMAT), as a part of its trace-evidence quality-assurance guidelines, has recommended a 12-month, full-time training program for inexperienced examiners[38]. The SWGMAT quality-assurance guidelines also include a proposed curriculum for such a training program.
2. Proficiency testing of forensic hair microscopists: SWGMAT has recommended that all trace evidence examiners (including hair microscopists) complete at least one proficiency test each year. SWGMAT has also proposed guidelines for the preparation, administration, and interpretation of trace-evidence proficiency tests[39].
3. Error rates of microscopical hair comparisons: further studies such as those of Wickenheiser and Hepworth[26], Peabody[27], and Deedrick et al.[35] should be conducted and published. Such publications will also expand the sparse scientific literature on this subject.
4. Opinions of hair microscopists: In light of the opinions offered in court by hair microscopists such as Joyce Gilchrist, standards for the interpretation of the results of microscopical hair comparisons should be developed.

Until these issues have been addressed and satisfactorily resolved, microscopical hair comparisons must be regarded by law-enforcement agencies and courts of law as merely presumptive in nature, and all microscopical hair comparisons must be confirmed by nuclear DNA profiling or mitochondrial DNA sequencing.
